# Yoga Programme for Type 2 Diabetes Prevention (YOGA-DP): A Qualitative Study Exploring the Trial Team’s Facilitators and Challenges in Conducting a Feasibility Trial in India

**DOI:** 10.1007/s13300-023-01450-0

**Published:** 2023-08-10

**Authors:** Pallavi Mishra, Sheila Margaret Greenfield, Tess Harris, Mark Hamer, Sarah Anne Lewis, Kavita Singh, Rukamani Nair, Somnath Mukherjee, Nikhil Tandon, Sanjay Kinra, Nandi Krishnamurthy Manjunath, Dorairaj Prabhakaran, Kaushik Chattopadhyay

**Affiliations:** 1 Centre for Chronic Disease Control, New Delhi, India; 2 Institute of Applied Health Research, University of Birmingham, Birmingham, UK; 3 Population Health Research Institute, St. George’s University of London, London, UK; 4 Division of Surgery and Interventional Science, Institute Sport, Exercise and Health, University College London, London, UK; 5 Lifespan and Population Health, School of Medicine, University of Nottingham, Nottingham, UK; 6 Bapu Nature Cure Hospital and Yogashram, New Delhi, India; 7 Department of Endocrinology, Metabolism and Diabetes, All India Institute of Medical Sciences, New Delhi, India; 8 Department of Non-Communicable Disease Epidemiology, London School of Hygiene and Tropical Medicine, London, UK; 9 Swami Vivekananda Yoga Anusandhana Samsthana, Bangalore, India

**Keywords:** Challenges, Facilitators, Prediabetes, Qualitative research, Semi-structured interviews, Trial, Type 2 diabetes mellitus, Yoga

## Abstract

**Background:**

In India, around 77 million people are at high risk of developing type 2 diabetes mellitus (T2DM). Yoga interventions can be effective in preventing T2DM. We conducted a feasibility randomized controlled trial (RCT) in India, and the intervention was the Yoga Programme for T2DM Prevention (YOGA-DP). This study aimed to identify and explore the facilitators and challenges in conducting the feasibility trial in India, and more specifically, to explore the perceptions and experiences of trial staff in relation to running the feasibility trial and Yoga instructors in relation to delivering the intervention.

**Methods:**

An exploratory qualitative study was conducted at two trial sites in India (Yoga centers in New Delhi and Bengaluru). Semi-structured interviews were conducted with ten participants (six trial staff and four Yoga instructors) to explore their perceptions and experiences related to the study’s aim. Data were analyzed using deductive as well as inductive logic and an interpretative phenomenological approach.

**Results:**

Feasibility-trial-related facilitators were useful participant recruitment strategies and help and support received from the trial coordination center. Intervention-related facilitators were strengths of the intervention content, structure, and delivery (including materials) and competencies of Yoga instructors. Feasibility-trial-related challenges were lack of awareness about T2DM among potential participants, stigma and fear associated with T2DM among potential participants, difficulties in explaining the research and obtaining written informed consent from potential participants, expectations and demands of potential participants and control-group participants, gender and language issues in participant recruitment, other participant recruitment-related challenges, issues in participant follow-up, and issues in data collection and trial documentation. Intervention-related challenges were the limited interest of participants in Yoga, participants’ time constraints on practicing Yoga, participants’ health issues hindered Yoga practice, beginners’ difficulties with practicing Yoga, participants’ demotivation to practice Yoga at home, issues with the Yoga practice venue, confusion regarding the intervention structure, issues with intervention materials, and the incompetence of Yoga instructors.

**Conclusions:**

The perceptions and experiences of trial staff and Yoga instructors helped us to understand the facilitators and challenges in running a feasibility trial and delivering the intervention for T2DM prevention, respectively. These findings and their suggestions will be used when designing the definitive RCT for evaluating YOGA-DP’s effectiveness, and may be helpful to researchers planning similar trials.

**Trial Registration Number:**

India (CTRI) CTRI/2019/05/018893.

## Key Summary Points

**Table:** 

In India, many people are at high risk of developing type 2 diabetes mellitus (T2DM). Yoga interventions can be effective in preventing T2DM.
An exploratory qualitative study was conducted in India as part of a feasibility randomized controlled trial (RCT), and semi-structured interviews were conducted with the trial team. The intervention was the Yoga Programme for T2DM Prevention (YOGA-DP).
The perceptions and experiences of trial staff and Yoga instructors helped us to identify and explore the facilitators and challenges in running a feasibility trial and delivering the intervention for T2DM prevention, respectively. These findings and their suggestions will be used when designing the definitive RCT for evaluating YOGA-DP’s effectiveness, and may be helpful to researchers planning similar trials.

## Introduction

In India, around 77 million people are at high risk of developing type 2 diabetes mellitus (T2DM), and these people have higher blood glucose levels than normal but lower than the established threshold for T2DM [1]. The major risk factors for T2DM include an unhealthy lifestyle (i.e., physical inactivity and an unhealthy diet), which is common among Indians [2]. Screening and delivering an effective lifestyle intervention is a cost-effective strategy for improving blood glucose levels and preventing T2DM among high-risk individuals [2].

Yoga, an ancient Indian mind–body discipline, is derived from the Sanskrit term ‘Yuj’, which means ‘to yoke’ or simply ‘to unite’. It is a union of the individual spirit with the universal spirit of God [3]. Traditionally, it has been classified as Japa Yoga, Karma Yoga, Gyana Yoga, Bhakti Yoga, Raja Yoga (or Ashtanga Yoga), Swara Yoga, Kundalini Yoga, and Nadi Yoga [3]. The Father of Yoga, Maharishi Patanjali, advocated Ashtanga Yoga or the eight steps of Yoga, namely, Yama (self-control), Niyama (discipline), Asana (Yogic poses), Pranayama (breathing practices), Pratyahara (withdrawal of the senses), Dharana (concentration), Dhyana (meditation), and Samadhi (complete realization) [3]. The Indian government is committed to and has prioritized the prevention and management of non-communicable diseases like T2DM through traditional Indian therapies like Yoga, and the Ministry of Ayush is dedicated exclusively to traditional Indian therapies [4]. The idea of the United Nations International Day of Yoga (21 June) was first proposed by the Indian government [5]. In the West, Yoga is considered a form of exercise, and some of the most common styles are Ashtanga Yoga, Iyengar Yoga, and Sivananda Yoga. However, no style is necessarily better than the other [6].

Previous systematic reviews have highlighted that Yoga interventions can play a vital role in preventing T2DM among high-risk individuals [7–10]. Yoga is a lifestyle intervention that includes low- and moderate-intensity physical activities and education on a healthy diet [11–13]. Yoga has been a part of the Indian culture, and thus Yoga interventions are more acceptable to Indians [11, 12]. Yoga requires a moderate level of guidance for participants [9] and is delivered gently [11]. Yoga is considered safe and can be practiced by people with multiple comorbidities [11, 13]. Another important benefit of Yoga is the low cost; it requires no or limited infrastructural support and can be practiced both outdoors and indoors [11].

The Yoga Programme for T2DM Prevention (YOGA-DP) was systematically developed [14]. YOGA-DP is a 24-week-long structured lifestyle education and Yoga-based exercise program. In the first 3 months, at least two YOGA-DP sessions per week are delivered to participants at the site. In the next 3 months, at least one session every 4 weeks is delivered to participants at the site. In addition, from the fourth month onwards, they are expected to have at least two unsupervised sessions per week at home. They are given intervention booklets, a Yoga video on a USB flash drive, a Yoga diary to record their practice, and a non-slippery Yoga mat. A feasibility randomized controlled trial (RCT) was conducted in India to determine the feasibility of undertaking a definitive RCT and to estimate important parameters needed to design the definitive RCT [15–17]. The definitive RCT will assess the effectiveness of the intervention in preventing T2DM among high-risk people in India. As part of the feasibility RCT, this study aimed to identify and explore the facilitators and challenges in conducting the feasibility trial in India, and more specifically, to explore the perceptions and experiences of trial staff in relation to running the feasibility trial and Yoga instructors in relation to delivering the intervention.

## Methods

An exploratory qualitative study was conducted to explore participants’ perceptions and experiences related to the study’s aim. The feasibility study protocol and other qualitative process evaluation work are published elsewhere [15, 18, 19]. Briefly, the feasibility study was carried out at two Yoga centers—one in northern India [Bapu Nature Cure Hospital and Yogashram (BNCHY), New Delhi] and one in southern India [Swami Vivekananda Yoga Anusandhana Samsthana (S-VYASA), Bengaluru]. Semi-structured interviews were conducted with two site investigators (responsible for leading the trial at the site), two trial coordinators [responsible for day-to-day trial coordination and data (case report form one) collection and entry at the site], two research assistants [responsible for data (case report forms two and three) collection and entry at the site], and four Yoga instructors (male and female; responsible for delivering the intervention at the site). In short, ten interviews were conducted (i.e., five per trial site).

Semi-structured interviews were conducted by a trained qualitative researcher, either face-to-face at the trial site or through telephone, by prior appointment from March to June 2020 [20, 21]. Using a predeveloped interview guide, interviews were conducted in English or Hindi, as preferred by trial staff and Yoga instructors. Interviews were digitally recorded with permission and transcribed verbatim by a professional transcriber at the trial coordination center [Centre for Chronic Disease Control (CCDC), New Delhi], and if needed, translated from Hindi to English by a professional translator at CCDC. Then, the qualitative researcher repeatedly listened to the interview recordings and added any missing data, corrected any inaccuracies, and anonymized the transcripts.

At the outset of data analysis, the qualitative researcher familiarized herself with the data by reading and re-reading the transcripts. Transcripts were coded using deductive as well as inductive logic and an interpretative phenomenological approach [22]. QSR NVivo 10 software was used for coding data, and summaries for each code were prepared [22, 23]. A priori themes were generated using the key questions in the interview guide. The qualitative researcher continuously discussed the process with the study investigators (including a senior qualitative researcher) and refined themes and sub-themes following feedback. The original data were continuously referred to to ensure that the perceptions and experiences of the participants were accurately and adequately presented. The emergent themes were captured after the first stage of free textual analysis to annotate any interesting or significant response from the participant. Later, these themes were organized in analytical or theoretical order to make sense of connections between themes [24]. All the quotes represented below are anonymized but attributed to a designation for trial staff and Yoga instructors and attributed to a sex in the case of Yoga instructors.

Ethics approval was obtained from the following research ethics committees: Faculty of Medicine and Health Sciences, University of Nottingham, UK (14-1805); CCDC, India (CCDC_IEC_09_2018); BNCHY, India (BNCHY/IEC/2/2019); and S-VYASA, India (RES/IEC-SVYASA/138/2018). The participant information sheet and consent form was shared with trial staff and Yoga instructors, and the qualitative researcher answered their questions if they had any. In the consent form, the participants were asked to provide written consent for the publication of verbatim responses. Before the interview, written informed consent was obtained from them to participate in the study and publish the findings. This study was in accordance with the Declaration of Helsinki.

## Results

Interviews with trial staff and Yoga instructors ranged between 32 and 77 min in length, with an average duration of 48 min. Presented data are divided into four analytical themes (i.e., feasibility-trial- and intervention-related facilitators and challenges) and 21 sub-themes (Table 1).

**Table 1:** Themes and sub-themes

Themes	Sub-themes
Facilitators	
Feasibility trial related	Useful participant recruitment strategies
	Help and support received from the trial coordination center
Intervention related	Strengths of intervention content, structure, and delivery (including materials)
	Competencies of Yoga instructors
Challenges	
Feasibility trial related	Lack of awareness about T2DM among potential participants
	Stigma and fear associated with T2DM among potential participants
	Difficulties in explaining the research and obtaining written informed consent from potential participants
	Expectations and demands of potential participants and control group participants
	Gender and language issues in participant recruitment
	Other participant recruitment-related challenges
	Issues in participant follow-up
	Issues in data collection and trial documentation
Intervention related	Limited interest of participants in Yoga
	Participants’ time constraints on practicing Yoga
	Participants’ health issues hindered Yoga practice
	Beginners’ difficulties in practicing Yoga
	Participants’ demotivation to practice Yoga at home
	Issues with the Yoga practice venue
	Confusion regarding the intervention structure
	Issues with intervention materials
	Incompetence of Yoga instructors

### Facilitators

This theme includes factors that helped trial staff to run the feasibility trial and Yoga instructors to deliver the intervention.

#### Feasibility-Trial-Related Facilitators

*Useful participant recruitment strategies*. Trial staff used various strategies to recruit participants. One of the most effective strategies was to organize screening camps at one location for a longer duration, and people in the vicinity became aware of the feasibility trial. Their previous contacts with people around the trial site, including door-to-door campaigns by them, facilitated the recruitment.

“Initially, we put up banners at all the public places that we know of. Secondly, we distributed pamphlets with newspapers. We also spoke to people whom we knew from our previous projects. Initially, we had these three strategies. There was one guy who used to sit at the hospital entrance every morning and randomly inform people about the study. The latter strategy was more successful … Apart from this, if there was any particular festive season like at that time it was Ganesh Chaturthi (Hindu festival), we used to put up banners in tents housing Ganesha’s idol. We used to put up our stall there and be present there.” (trial coordinator)

“He (trial coordinator) has developed many contacts for a long time and has participated in previous projects. We called them (people in the vicinity) and gave them some information that such a project is going on here, and if someone does not have diabetes and does not know about their (blood) sugar level, they come to us for free testing. So, many people, who were part of previous projects, came along with their relatives, and we were able to recruit in large numbers from there.” (research assistant)

“ … we started going door-to-door. There was one benefit, we also took a few students as a part of this project, just for the sake of helping us with manpower, to distribute the pamphlets that we had created for YOGA-DP in terms of screening and just telling them (people)—‘OK, there will be screening, and you can come and get your blood checked and other things’.” (research assistant)

The involvement of an influential and respectable local person also facilitated the recruitment. People got motivated to appear for the screening to participate in the feasibility trial, which otherwise was difficult.

“So, I used to visit the trial site and try to identify one person who is a well-known person there. For example, in one of the villages, one of the sites, we had one person, who was like the head of the village or something like that.” (research assistant)

One of the trial sites also recruited a female clinician who visited the community along with the trial coordinator so that she could address women’s concerns and encourage them to participate in the feasibility trial.

“We knew that this male and female thing would be an issue, so we always had a female doctor with us, and we never went and approached a person alone, because we do not want any problems there.” (research assistant)

*Help and support received from the trial coordination center*. The help and support received from the trial coordination center throughout the feasibility trial facilitated the whole study process at the site, including randomization.

“I used to talk to you (qualitative researcher) and the randomization officer in CCDC. (Name) ma’am used to call us for the randomization part. It was perfect. I faced no problem there.” (research assistant)

#### Intervention-Related Facilitators

*Strengths of intervention content, structure, and delivery (including materials)*. The content and structure of the intervention were appreciated by Yoga instructors. For example, loosening exercises prepared participants for challenging Yogic practices, which they could not do otherwise.

“Firstly, your study (intervention) has been designed really well. Since I have participated in many such studies before, so I was searching for something new in this study. You are focusing on people with prediabetes. With the help of this study, I started motivating people that … it will give you lifelong relief.” (male Yoga instructor)

“There were some exercises, which they (participants) were not able to do at all and felt pain, but because of our loosening exercises, it became easy for them. I had to give them a demo, in the beginning, to show them how to do it.” (male Yoga instructor)

The delivery of the intervention was well received; for example, providing separate Yoga sessions for men and women, attending Yoga sessions at their convenience, practicing Yoga as per their capacity, and receiving regular calls for support and troubleshooting their problems.

“We used to have little modification for the group with males and females. In a room, we had both genders together, so we used to make them stand on opposite sides, turn around the other way and give instructions so that they did not feel awkward during any Yogic practice, thinking if someone was seeing or not. So, in that way, it was fine.” (female Yoga instructor)

“But if they (participants) missed the classes, as per the guidelines, the compensatory classes were taken in the same week. If they were not able to come, they used to call and inform—‘today it is not possible because I have this and this work’. So, we used to say—‘please sort it out and please attend tomorrow’s class’.” (female Yoga instructor)

“I did not force them (participants) because they all had different levels of flexibility and anatomy. So, we cannot force them and push everyone to do the same. So, I used to tell them to try to bend and touch your knees with your hands.” (female Yoga instructor)

“I used to call my participants, to remind and guide them about their home sessions. Sometimes, I used to leave a message for them and not call them. I used to do this daily for many participants.” (male Yoga instructor)

The intervention materials (booklets and a video for participants and a manual for Yoga instructors) were found to be useful. For example, participants used the Yoga video during home-based unsupervised sessions. Yoga instructors studied Yogic practices as part of their academic training, and the manual helped them to deliver the exact intervention.

“The whole protocol is given in the second one (Yoga-DP booklet part II) in a very good manner, which is a very good thing. The first one (Yoga-DP booklet part I) has information about what kind of diet one should take and avoid … The booklet has been given a lot of thought while compiling it. So, I did not find anything wrong with it.” (female Yoga instructor)

“The video in the pen drive was good, and participants liked it. They said that by watching the video even their family members are practicing Yoga. Some said that they do it along with their family members. So, the pen drive was of great help to them.” (male Yoga instructor)

“It (manual) was very good. When we were talking with the (local) PI of the study, he told us that he along with many others have collaborated to develop it. We also did a practical on that.” (female Yoga instructor)

*Competencies of Yoga instructors*. Yoga instructors were approachable and regularly interacted with participants and solved their problems, which created a positive environment during Yoga sessions and motivated participants to attend.

“I used to talk to them (participants) regularly like friends. They used to share their problems with me, so they used to come regularly because of this. I was friendly with them, and they would listen to me because of that.” (female Yoga instructor)

“A Yoga instructor or therapist should have a friendly nature, and he should always keep a smile on his face because everyone is looking at him all the time. If they go to conduct a session with an unpleasant face, then participants will get demotivated.” (male Yoga instructor)

“I did not call them (participants) regularly, but I was connected to them via WhatsApp. I used to message them, and they used to reply to me with good morning, good night, and many other things. And sometimes on the call, we used to talk about different things.” (female Yoga instructor)

Yoga instructors used to ask participants about the sequence of Yogic practices to help them memorize and adhere to the sequence.

“ … when they (participants) came for sessions in the hospital, I made them memorize everything by asking them like which Yogic practice comes next? And, then I used to ask them to do it in front of me.” (female Yoga instructor)

### Challenges

This theme includes factors that hindered trial staff to run the feasibility trial and Yoga instructors to deliver the intervention.

#### Feasibility-Trial-Related Challenges

*Lack of awareness about T2DM among potential participants*. Trial staff shared the feasibility trial-related information with potential participants; however, people felt that T2DM would not negatively impact their health and thus decided not to come for the blood test and participate in the trial.

“ … many ladies and gents of middle age group used to come, and we used to request them and say, ‘if you do not know your (blood) sugar level, you can get it tested if you want to’. After getting all this information and even after requesting them, it used to happen sometimes that they were not even interested and used to say, ‘these things (T2DM) do not cause much of a problem’.” (research assistant)

*Stigma and fear associated with T2DM among potential participants*. The stigma and fear associated with T2DM prevented potential participants from enrolling in the feasibility trial. They stopped interacting with trial staff after knowing their fasting blood glucose levels.

“One thing I found is that there is a stigma in the society or the community relating to being diagnosed with diabetes. I have heard a lot from one of the sites about this place which is quite close to our campus. It is a village. When we did the initial screening, we told them (some of the people) that their FBS (fasting blood sugar) was high, and then they were completely scared and stopped talking to us. They never came back. I think some people are scared, and they think diabetes is fatal and they are going to die.” (research assistant)

*Difficulties in explaining the research and obtaining written informed consent from potential participants*. Trial staff found it challenging to explain the technical aspects of the feasibility trial to potential participants in a way that they would understand, and this negatively affected the recruitment.

“For example, if you say, we are going to draw blood, we are going to do this test, HbA1c (glycated hemoglobin) or FBS (fasting blood sugar) or whatever and Yoga or control group, whichever it may be, will be done. So, they (potential participants) do not understand this concept and how it works. Then, they do not participate if they do not understand.” (research assistant)

A large number of people were coming to the screening camps, and the participant information sheet took a long time to read and explain to each potential participant.

“You people told us that we had to take the (written) informed consent of everyone who had agreed to the preliminary test. The more challenging part was reading PIS (participant information sheet) and explaining it to them.” (trial coordinator)

People were not ready to sign the consent form as they could not trust the research process. A lack of trust among potential participants negatively affected the recruitment.

“Most of the problems arose out of their (potential participants’) hesitation to sign the consent form. They used to ask—‘if you work in the health sector and want to test blood glucose levels, then what’s the need of our (signed) consent for it?’.” (research assistant)

*Expectations and demands of potential participants and control group participants*. Despite being an RCT, potential participants were expecting to receive the intervention. Even after randomization, control group participants insisted on joining the intervention group, and it was difficult to convince them to stay in the control group.

“We stay around 30 km from Bangalore city, and for all our projects, we conduct some camps, publicize, inform patients coming to our hospital, etc., to volunteer. So, this being a truly randomized controlled trial, one challenge that we faced was like all those who came to our campus were expecting Yoga. So, there was no way we could randomize them.” (site investigator)

“About the intervention, I would like to say that, at the time of recruitment, most people wanted to be part of the intervention. After the glucometer test, when they used to take information from us and when we used to tell them that there is Yoga for people with prediabetes, they didn’t want to listen to the second part any further. Most of the people requested to be placed in this group. And some people even backed out after we told them that it is not up to us which group they are placed in.” (research assistant)

Control group participants requested T2DM prevention medicine as they felt that the advice provided to them in the leaflet would not help prevent T2DM.

“Some (control group) participants, who used to come with their relatives, requested us to provide medicines as well. Some people thought that we had just recruited them in the control group and told them about the diet chart, which was insufficient. They believed that they should be given medicines as well.” (research assistant)

*Gender and language issues in participant recruitment*. It was difficult for male trial staff to reach out to women for participation in the feasibility trial. Trial staff had to convince male family members to allow women to participate in the trial.

“There were many cases where I faced problems in coordinating. There was one lady whose mother was not picking up the call, and sometimes her husband or son were picking up the calls. And they used to avoid the call after listening to my or sir’s voice. So, we tried to call her for the test somehow, as there was also an issue. We even went to her door, shared the consent form on WhatsApp, and requested her to read it once. She then searched it on Google.” (research assistant)

One of the research assistants could not speak to potential participants during the recruitment as he did not know the local language.

“I think language is critical. One aspect that we found has a major role in convincing them (potential participants) or conveying the message—‘OK, this is the project’. At times, some people did not speak the language I know or the people around me. So, it became quite challenging to convince them. So, that was one challenge.” (research assistant)

*Other participant recruitment-related challenges*. Participant recruitment was a time-consuming task, which negatively impacted other research-related activities of trial staff.

“At times, when things start piling up, all of a sudden, it becomes challenging. For example, initially, when we had to screen many people, we had to spend much time traveling, and that was quite—I would not say it was challenging, rather it was hectic.” (research assistant)

People visiting the screening camps lied about the duration of their fast to have a free blood glucose test.

“The major challenge was regarding fasting sugar level. If you ask someone, ‘when did you have your meal yesterday?’ I think 40% of people tell a random time. Even if he ate at 11 PM, he will still say that he ate around 8–9 PM. These 3 h difference matters a lot for you.” (trial coordinator)

*Issues in participant follow-up*. In both groups, participant follow-up was not easy—sometimes, due to limited interest in the study. Follow-ups were even more difficult in the control group participants as tracing them after 6 months of baseline data collection was difficult. In addition, the COVID-19 pandemic and the related national lockdown in India added more to this problem towards the end of the study.

“I do not know. I mean there is nothing that we can do, I think. It was just the response of people. Somehow, I feel that they were interested in exploring Yoga, they were interested in blood sugar status, they were just, I felt a little bit more motivated to be a part of the study, and after whatever group Yoga or control they were in, after 6 months, to get their response again, somehow, they did not respond.” (trial coordinator)

“Most problems were related to the control group, and we faced problems in its coordination. Because we were in touch with all the intervention group participants, as they were coming for the Yoga sessions, their classes were going on as per the schedule at their preferred time … If we talk about the control group, some people had even forgotten that they had their testing done. So, we were trying to call everyone.” (research assistant)

“When this happened—the issue with the virus (COVID-19), it became quite a significant hurdle for us to contact and reach out to participants. It posed a threat for both participants and us, in going out when there is a lockdown and when there is police monitoring. We were questioned during one of the follow-up visits.” (research assistant)

*Issues in data collection and trial documentation*. Trial staff struggled to complete the case report forms (CRFs) due to several reasons. For example, a few questions in the CRFs were unclear to trial staff, and they struggled to explain some of the questions to participants. Participants were hesitant or struggled to recall (e.g., fruit and vegetables intake) or express (e.g., EuroQol-5D for assessing health-related quality of life).

“I did not understand the part where you had photos of fruit and vegetables. I did not get why those photos were put there. I mean its basis. I feel that it would have been easier to ask the question—‘what did you eat?’.” (trial coordinator)

“While explaining it (questions), I sometimes found it hard depending on the educational level. It’s not necessary that less educated people needed more explanation … If the same was done in the UK, I am sure that even the school-going kids there are much more articulate, and then we think, how we use language here.” (trial coordinator).

“They (participants) were hesitant. I don’t know what the reason was, but somehow it was easier to get the people for blood samples than to answer the CRF. There were some questions in the CRF which as you know—as from my experience I feel that we are—we Indians are less articulate when it comes to expressing ourselves.” (trial coordinator)

Even after the feasibility trial initiation training, it was difficult for trial staff to remember all the trial-related forms and documents and the process to complete these. They had to frequently call the trial coordination center for help and felt the need for refresher training. They also suggested that the trial initiation training should have been spread across several days, as it was information overload in a day.

“It would have been better if you could have provided more elaborate training about these at the beginning. We sometimes could not understand if we had to fill it out or not, and we consulted you repeatedly. So, it would have been better if we were briefed more about it.” (trial coordinator)

“There are few cases in which one cannot understand everything in one go, and so, it will be better if this (training) is repeated 1–2 times in between. Or, it can also be so that in starting weeks, this can be repeated 2–3 times, making it more helpful.” (research assistant)

#### Intervention-Related Challenges

*Limited interest of participants in Yoga*. Participants were more interested in the diet counseling part than in the Yoga sessions. They believed that they could practice Yoga independently and were not ready to spend time on it.

“They (participants) mostly spoke to me about the diet. They used to ask me about various diets. They used to say that they knew the exercises (Yoga) and would be able to do them on their own. They did not give themselves as much time as they could.” (female Yoga instructor)

*Participants’ time constraints on practicing Yoga*. Although we reimbursed some of the local travel costs of participants or arranged the Yoga sessions locally, it was difficult for participants to allocate 75 min to practice Yoga due to their household and professional responsibilities, especially poor people and women. They were tired after a long working day.

“But for people in rural areas, the day starts much early. They wake up early as they have a lot of work to do, so if you ask them to do it, it is difficult for them to do it. So, the duration between 45 min to 1 h is more than enough. More than that is extra for them and burdens them.” (female Yoga instructor)

“People from different economic statuses and gender have different responsibilities. For example, a daily wage earner has to work daily to earn money. So, it becomes difficult for him to come into such a scenario. And also, some people are well-established but are so lazy that they do not want to wake up in the morning.” (male Yoga instructor)

*Participants’ health issues hindered Yoga practice*. Participants had difficulties practicing Yoga due to other health conditions. Women missed Yoga sessions during menstruation due to the associated physical discomfort.

“Yes, I know a participant who has slipped disc problem. She cannot do Surya Namaskar at all. So, it became challenging for us to make her do Surya Namaskar, and we could not even push her to do it. There were many Yogic practices which she couldn’t do like Paschimottanasana, Surya Namaskar, and 1–2 more.” (female Yoga instructor)

“The ladies face the problem at the time of the menstrual cycle. So, we had two classes per week, and some of them missed it due to their menstrual cycle date.” (male Yoga instructor)

*Beginners’ difficulties in practicing Yoga*. Beginners had difficulties in practicing some of the Yogic practices and initially had body aches, which were normal.

“In the sessions, there was Shalabhasana, and they (participants) were finding it difficult to do a few steps in Surya Namaskar because they were very new to it. They did not do Yoga for many years. The age group we had was in the 30–40 years club. So, it was a little difficult for them to adjust to all the Yogic practices and do it properly.” (female Yoga instructor)

“Yes, when they (participants) started initially, they faced strain issues, pain issues, etc. which is normal for anyone who has done no exercise at all. We know it is normal, nothing to worry about, and it will get fine after 2–3 sessions.” (male Yoga instructor)

*Participants’ demotivation to practice Yoga at home*. Participants requested Yoga instructors to visit them at home and supervise the home-based Yoga sessions. They felt lazy and demotivated to practice Yoga at home.

“They (participants) do not want to do Yoga at home. They ask me to come and teach them Yoga daily. They say that they become lazy at home, and if someone teaches them, they are attentive. They say that they are not able to do it at home.” (male Yoga instructor)

*Issues with the Yoga practice venue*. At one of the trial sites, the room for Yoga sessions was not well ventilated, and thus it was difficult for participants to practice Yoga during the summer months.

“The venue was perfect for the winter season. They (BNCHY) had prepared structures out of soil/mud, which was very good. The only issue was the cross-ventilation of air, due to which participants faced some problems. They sometimes used to feel the heat.” (female Yoga instructor)

A change in venue during the feasibility trial was a challenge. It was difficult to find a new place and convince participants to come to the new venue.

“But after a few days, the venue owner told us that they wanted to rent it out so we should change the venue. So, there was some pressure on us to manage that situation. It is difficult to find an empty warehouse in a city like Bangalore.” (male Yoga instructor)

*Confusion regarding the intervention structure*. Although the intervention structure was clearly described in the manual for Yoga instructors, there was some confusion regarding the structure and, more specifically, the number of Yoga sessions per week.

“Also, you had a clause that Yoga sessions should be conducted twice a week and not one session is held per week. So, these are specific things you will have to emphasize while explaining and how to calculate these. There is one thing that even I forgot to ask you. Suppose a person is coming only once a week. For example, if a person is a government employee and can come only once a week, i.e., on Sundays, what will you do in that case?” (trial coordinator)

*Issues with intervention materials*. Although the intervention was developed in consultation with people, participants struggled to understand the Yoga booklet. Yoga instructors, as expected, helped in making them understand. Participants struggled to use the Yoga video at home for unsupervised sessions as it was slow and long.

“The challenge was, in the beginning, to make them (participants) understand a few Yogic practices in the way we were delivering the instructions. Making it simpler for them was difficult as we were asked to stick to the rules and instructions given in the book. However, we used to make them understand a few words after the class or during the class so that it would be easy for them to learn new words and practices.” (female Yoga instructor)

“One thing that I would like to add is that the video was long according to participants. The audio and playback were very slow. So, participants struggled to follow it at home. Participants used to complain about that to me.” (female Yoga instructor)

Regular completion of the Yoga diary at home for unsupervised sessions was a challenging task for participants, and they struggled to record the exact number of minutes for which they did each Yogic practice.

“As gathered from participants, the diary entry part was somewhat difficult for them to do. They found it difficult to fill it up again and again.” (male Yoga instructor)

*Incompetence of Yoga instructors*. At times, trial staff felt that Yoga instructors at the site were incompetent at understanding participants’ needs, including their health problems.

“Yes, there was some inexperience. For example, in one case, a lady encountered some problem, but the Yoga instructor did not take it seriously for 2–3 days. When I developed doubt, I asked her (Yoga instructor) to call her again because she had developed some medical problem. When she called her, she told her that there was some medical problem with her. So, the team was inexperienced.” (trial coordinator)

## Discussion

We identified and explored several facilitators and challenges in running the feasibility trial and delivering the intervention as perceived and experienced by trial staff and Yoga instructors, respectively. Feasibility-trial-related facilitators were useful participant recruitment strategies and help and support received from the trial coordination center. Intervention-related facilitators were strengths of the intervention content, structure, and delivery (including materials) and competencies of Yoga instructors. Feasibility-trial-related challenges were lack of awareness about T2DM among potential participants, stigma and fear associated with T2DM among potential participants, difficulties in explaining the research and obtaining written informed consent from potential participants, expectations and demands of potential participants and control group participants, gender and language issues in participant recruitment, other participant-recruitment-related challenges, issues in participant follow-up, and issues in data collection and trial documentation. Intervention-related challenges were the limited interest of participants in Yoga, participants’ time constraints on practicing Yoga, participants’ health issues hindered Yoga practice, beginners’ difficulties in practicing Yoga, participants’ demotivation to practice Yoga at home, issues with the Yoga practice venue, confusion regarding the intervention structure, issues with intervention materials, and the incompetence of Yoga instructors. These findings and their suggestions will be used when designing the definitive RCT for evaluating YOGA-DP’s effectiveness.

Experiences gained from previous studies and existing contacts with local leaders and people facilitated participant recruitment. Another clinical trial also emphasized the importance of existing contacts with local people [19]. Trial staff reported that obtaining written informed consent from potential participants was one of the most challenging parts of the recruitment process, and people were hesitant to sign the consent form. A previous study conducted in Peru, another low- and middle-income country, explored the challenges in obtaining informed consent from participants—they were afraid of losing their land property by signing any paper and preferred to give verbal consent [25]. Some trial staff mentioned language barriers in communications with people and the negative impact they had on participant recruitment. This is a well-known issue in research involving people [19]. Similar to the finding of a previous systematic review [26], the participant follow-up was challenging, and the reason for the loss to follow-up among control group participants was the long duration between baseline data collection and follow-up, with limited contact between trial staff and control group participants. Although trial staff members were given training before starting the feasibility trial, they found it difficult to remember all aspects of the study. Another clinical trial highlighted similar difficulties faced by data collectors despite training [27].

To the best of our knowledge, this was the first qualitative study exploring the facilitators and challenges in conducting a feasibility trial in India and, more specifically, exploring the perceptions and experiences of trial staff in relation to running the feasibility trial and Yoga instructors in relation to delivering the intervention. Trial staff with different responsibilities were interviewed to provide a complete picture of running a feasibility trial. Some of the study findings were perceptions developed (assumptions made) by trial staff and Yoga instructors about people and participants, and these should be interpreted with caution (for example, the stigma and fear associated with T2DM among potential participants). Our qualitative process evaluation work with people and participants is published elsewhere [18, 19]. The qualitative researcher was also the project manager based at the trial coordination center, and this could have influenced the study findings. However, there was a positive side to this approach as well—she had detailed knowledge of the project, which helped when probing and discussing minute details with interviewees.

In conclusion, the perceptions and experiences of trial staff and Yoga instructors helped us to understand the facilitators and challenges in running a feasibility trial and delivering the intervention for T2DM prevention, respectively. These findings and their suggestions will be used when designing the definitive RCT for evaluating YOGA-DP’s effectiveness, and may be helpful to researchers planning similar trials.
